# Use of cinacalcet and sunitinib to treat hypercalcaemia due to a pancreatic neuroendocrine tumor

**DOI:** 10.1590/2359-3997000000291

**Published:** 2017-09-04

**Authors:** Hernan Valdes-Socin, Matilde Rubio Almanza, Mariana Tomé Fernández-Ladreda, Daniel Van Daele, Marc Polus, Marcela Chavez, Albert Beckers

**Affiliations:** 1 Service d’ Endocrinologie CHU de Liège Belgium Service d’ Endocrinologie. CHU de Liège, Belgium; 2 Servicio de Endocrinología y Nutrición Hospital Universitari i Politècnic La Fe Valencia Spain Servicio de Endocrinología y Nutrición, Hospital Universitari i Politècnic La Fe, Valencia, Spain; 3 Unidad de Gestión Clínica de Endocrinología y Nutrición Hospital Universitario de Valme, Área de Gestión Sanitaria Sur de Sevilla Spain Unidad de Gestión Clínica de Endocrinología y Nutrición, Hospital Universitario de Valme, Área de Gestión Sanitaria Sur de Sevilla, Spain; 4 Service de Gastroentérologie CHU de Liège Belgium Service de Gastroentérologie, CHU de Liège, Belgium; 5 Department of Medicine, Division of Hematology CHU de Liège Liège Belgium Department of Medicine, Division of Hematology, CHU de Liège, Liège, Belgium

## Abstract

Neuroendocrine tumors (NETs) can secrete hormones, including ectopic secretions, but they have been rarely associated with malignant hypercalcemia. A 52-year-old man with a history of diabetes mellitus was diagnosed with a pancreatic tumor. A pancreatic biopsy confirmed a well-differentiated pancreatic NET (pNET). The patient subsequently developed liver metastasis and hypercalcemia with high 1,25 OH vitamin D and suppressed parathyroid hormone (PTH) levels. Hypercalcemia was refractory to chemotherapy, intravenous saline fluids, diuretics, calcitonin and zoledronate. Cinacalcet administration (120 mg/day) resulted in a significant calcium reduction. Hypocalcemia was observed when sunitinib was added three months later and cinacalcet was stopped. Subsequently, the calcium and PTH levels normalized. After six months, we observed 20% shrinkage of the pancreatic tumor and necrosis of a liver metastasis. Cinacalcet is an allosteric activator of the calcium receptor agonist, and it is used for severe hypercalcemia in patients with primary (benign and malignant) hyperparathyroidism. In this patient, cinacalcet demonstrated a calcium lowering effect, normalized hypophosphatemia, and improved the clinical condition of the patient. The mechanism through which cinacalcet improved PTH-rp mediated hypercalcemia is still unclear, but studies have suggested that a potential mechanism is the activation of calcitonin secretion. Sunitinib is an oral multi-targeted tyrosine kinase inhibitor used to treat advanced pNETs. The hypocalcemic effects of sunitinib have not been previously described in a patient with pNET. Here, we report for the first time the successful combination of cinacalcet and sunitinib in the treatment of a pNET patient presenting with malignant hypercalcemia.

## INTRODUCTION

Hypercalcemia of malignancy (HCM) develops as a paraneoplastic process in several types of cancers, such as lymphoma, breast cancer, and lung cancer (1), but it has rarely been described in neuroendocrine tumors (NETs) (1-3). In the setting of neuroendocrine tumors (NETs), HCM is associated with advanced disease, poor prognosis, and decreased survival rates (4,5). The most common cause of HCM is the tumor secretion of parathyroid hormone related peptide or PTH-rp (PTH-rp-oma) (2,5). Pancreatic NETs are typically pluripotent, and they have the ability to produce several types of hormones, including PTH-rp and calcitonin (2-6). When feasible, primary tumor surgery should be performed to normalize hypercalcemia. Unfortunately, the conventional hypocalcemic treatment options (e.g., bisphosphonates, corticosteroids, diuretics, hyper hydration) do not seem to improve patient survival in these cases. Here, we report, for the first time, the successful combination of cinacalcet and sunitinib in a pNET patient presenting with malignant hypercalcemia. In this case, in the setting of pancreatic NET, hypercalcemia associated with hypophosphatemia and suppressed PTH levels were highly likely due to PTH-rp secretion, although serum PTH-rp levels could not be measured.

## CASE REPORT

**Figure 1:**
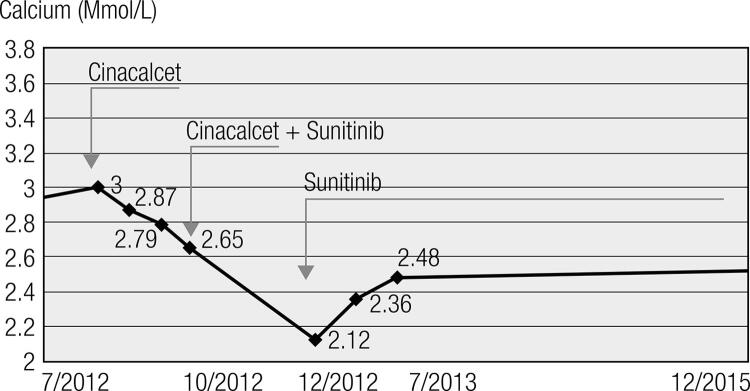
Serum calcium levels at the time of diagnosis, during the cinacalcet treatment (Mimpara© 120 mg/day PO), and during the combined treatment with Mimpara (120 mg/day) and sunitinib (Sutent© 35.7 mg/day). Cinacalcet was stopped when the calcium levels reached 2.12 mmol/L. The red line represents the upper normal calcium levels. The patient, whose calcium parameters remain normal, is currently being treated with Sutent only.

A 52-year-old-man with a history of diabetes mellitus and smoking presented with abdominal pain and asthenia for one year. Abdominal computed tomography (CT) revealed a 15 cm mass involving the pancreas and the retroperitoneum, with splenic and hepatic carcinomatosis. The histology of the pancreatic lesion showed a well-differentiated pNET positive for AE1/AE3, synaptophysin, and CD56. Chromogranin immunostaining was negative. The Ki-67 labelling index was 2%. Somatostatin-receptor scintigraphy (octreoscan) detected areas of pathologic uptake in the liver and pancreas, whereas bone scintigraphy did not reveal any skeletal metastatic deposits. Hypercalcemia was diagnosed with calcium and ionized calcium levels of 3.54 mmol/L (2.15-2.6) and 1.55 mmol/L (1.14-1.3), respectively. Other bone metabolism abnormalities included hypophosphatemia levels of 0.42 mmol/L (0.74-1.51), PTH levels of < 4 ng/mL (12-58), 1-25 OH vitamin D levels of 100 pg/mL (< 85), 25 OH vitamin D levels of 9 ng/ml (> 30) and calcitonin (as a tumor maker) levels of 1116 ng/mL (< 10). The chromogranin A level was 26.9 UI/L (< 23). Urine analysis showed hypercalciuria and hyperphosphaturia. A bone density analysis showed mild cortical and femoral osteopenia. Because surgery was not feasible, the patient underwent treatment with several cycles of streptozotocin-adriamycin and FOLFOX. The tumor mass and calcium levels were partially controlled (2.61 mmol/L), whereas the PTH concentration remained low (19 ng/mL). Three months later, the patient’s calcitonin levels were 29 ng/mL, and his calcium level increased again (2.94 mmol/L), whereas his PTH level was < 2 pg/mL. Treatment with octreotide LAR 30 mg sc every 4 weeks was introduced for 3 months, without any remarkable clinical impact. Hypercalcemia (total calcium 3.17 mmol/L) was refractory to intravenous saline fluids, diuretics, recombinant calcitonin, and zoledronate. Therefore, compassionate treatment with oral cinacalcet (120 mg/day) was attempted. The patient’s calcium level gradually decreased from 3.17 to 2.87 mmol/L and later to 2.65 mmol/L (Figure 1). The phosphatemia normalized from 0.42 mmol/L to 0.84 mmol/L during the cinacalcet treatment. PTH, 1,25 OH vitamin and calcitonin levels, as well as the tumor size, remained unchanged. After a significant clinical improvement following a three months of cinacalcet treatment (Mimpara^©^ 120 mg per day), sunitinib was added (Sutent^©^ 35.7 mg per day) for tumor control (Figure 1). Both drugs were well tolerated, without any side effects.

One month after beginning the combined treatment, the patient’s calcium level decreased to 2.12 mmol/L. Because of the hypocalcemia, the PTH level increased to 78 pg/mL, requiring discontinuation of cinacalcet. The calcitonin and 1,25 OH vitamin D levels normalized, whereas the positron emission tomography (PET) scan revealed a 20% shrinkage of the pancreatic tumor and necrosis of a metastasis in the VII hepatic segment following the cinacalcet and sunitinib treatment. Currently the patient is alive and still being treated with sunitinib. According to the RECIST (Response Evaluation Criteria in Solid Tumors) criteria, the patient has had stable disease for the last four years.

## DISCUSSION

HCM occurs in approximately 30% of all cancer patients. HCM may be caused by different mechanisms, including the production of parathyroid hormone-related peptide (PTH-rp), which is the most frequent cause (1-4), and/or 1,25 OH vitamin D by tumor cells. In addition, ectopic PTH secretion can rarely occur (1-4). NETs often produce ectopic hormone secretions, but they are rarely associated with HCM (2,5). In this case, an osteolytic lesion and a PTH-dependent mechanism of hypercalcemia were excluded. Unfortunately, we could not determine the PTH-rp levels of our patient, and no tumor specimen was available for PTH-rp studies. Therefore, it remains unproven whether PTH-rp hypersecretion was (at least partially) responsible for the hypercalcemia in this case.

Several treatment options are available to manage hypercalcemia in cancer patients (5-9). The extracellular volume must be restored with intravenous saline fluids because patients are frequently volume depleted (1). In addition, high diuretic doses can increase urinary calcium excretion levels (7). In addition, gallium nitrate, calcitonin, and hemodialysis have been used to treat cancer-related hypercalcemia (3). Glucocorticoids are frequently used to treat hypercalcemia in hematological malignancies. Intravenous bisphosphonates can be effective in treating HCM, and they have demonstrated extended durations of action and low rates of acute phase reaction symptoms (1-3). Denosumab, a human monoclonal antibody binding RANKL that inhibits osteoclast maturation and activation, is used to treat osteoporosis. Denosumab has been approved to treat HCM in the United States of America (USA), and it should be used in bisphosphonate resistant cases. It has been used to treat bisphosphonate-refractory HCM (8).

Cinacalcet is an oral drug that acts as a calcimimetic by activating the calcium-sensing receptor (CaSR). It is used to treat secondary hyperparathyroidism in chronic kidney disease and for severe hypercalcemia in patients with primary hyperparathyroidism who are not suitable candidates for parathyroidectomy (9). It is also indicated for the treatment of hypercalcemia in patients with parathyroid carcinoma. CaSR binding by cinacalcet results in decreased PTH secretion and synthesis, and it inhibits kidney 1,25 vitamin D synthesis and calcium reabsorption; in addition, it seems to have an anabolic effect on bone formation (9). Possibly because of high levels of a putative PTH-rp like peptide, the synthesis of 1,25 vitamin D was not affected in the above described patient during the cinacalcet treatment; however, it normalized when the patient was administered sunitinib. In murine models of Leydig cell and colon tumors, cinacalcet attenuated hypercalcemia without affecting the synthesis of PTR-rp mRNA by the tumor. This effect occurred in a dose-dependent manner independently from the PTH-rp administration in parathyroidectomized animals (10). Furthermore, these studies suggest that cinacalcet mediates the reduction in calcium levels, at least partially, by stimulating the release of calcitonin by C-cells. Another possible mechanism yet to be investigated could be the calcium lowering effect of cinacalcet through enhanced renal excretion of calcium (10).

Hypophosphatemia is also related to HCM, namely the decrease of renal reabsorption of phosphates, likely due to a PTH-rp-induced effect. In our case report, cinacalcet normalized low phosphates levels, as previously described in mouse models of HCM treated with cinacalcet. This effect has been attributed to an inhibitory effect of calcimimetics on phosphaturic hormones such as FGF-23 (9,10).

To the best of our knowledge, the hypocalcemic effects of cinacalcet have never been documented in pancreatic neuroendocrine tumor patients. The clinical case we have described is the second case in the literature in which cinacalcet was used successfully to treat refractory HCM. A previous report described a 57-year-old male with hypercalcemia and a pulmonary tumor secreting PTH-rp (11). In that case, the patient’s calcium and PTH-rp levels decreased during combined chemotherapy and cinacalcet monotherapy. When hypercalcemia recurred after the fourth chemotherapy cycle, cinacalcet monotherapy induced a consistent decline in PTH-rp levels, thus preventing a further increase in serum calcium levels (11).

Sunitinib is an oral, multi-target, tyrosine kinase inhibitor used to treat GIST, advanced renal cell carcinomas, and advanced pancreatic neuroendocrine tumors. A calcium-lowering effect of this drug was observed in a case of metastatic renal cell carcinoma and paraneoplastic hypercalcaemia (12). The authors observed that there was no reported case of paraneoplastic hypercalcemia recovery with targeted therapy until their report. We are unaware of any report, thus far, that describes the hypocalcemic effect of sunitinib in pNET patients.

In the clinical case reported here, the combined use of cinacalcet and sunitinib decreased the patient’s serum calcium levels to low-normal levels and normalized the PTH concentration. Although chemotherapy initially controlled the calcium levels at upper normal levels, the combined treatment induced a strong calcium lowering effect twice. After the commencement of combined cinacalcet + sunitinib treatment, the calcium levels were significantly lower compared to when the patient was undergoing cytotoxic chemotherapy. Moreover, the combined cinacalcet + sunitinib treatment resulted in a dramatic drop of calcium levels (i.e., hypocalcaemia) and induced the occurrence of secondary hyperparathyroidism.

## CONCLUSIONS

This case report describes the unusual association of malignant pNET-associated hypercalcemia, high 1,25 OH vitamin D, and high calcitonin levels. Several treatments options for the management of hypocalcaemia have been unsuccessfully attempted, finally leading to the compassionate use of cinacalcet. Cinacalcet demonstrated a definite calcium lowering effect and improved the clinical condition of the patient. Thus, we believe that cinacalcet can enrich the pharmacological armamentarium for the treatment of HCM. Moreover, sunitinib helped to normalize the patient’s calcium and calcitonin levels, with modest tumor shrinkage. The precise mechanism of the calcium lowering effect of sunitinib remains to be elucidated. In conclusion, here, we report for the first time the successful use of cinacalcet and sunitinib in the management of pNET-associated HCM.
